# Alcohol Consumption and Mortality: The Khon Kaen Cohort Study, Thailand

**DOI:** 10.2188/jea.JE20130092

**Published:** 2014-03-05

**Authors:** Siriporn Kamsa-ard, Supannee Promthet, Sarah Lewington, Julie Ann Burrett, Paul Sherliker, Supot Kamsa-ard, Krittika Suwanrungruang, Donald Maxwell Parkin

**Affiliations:** 1 Department of Biostatistics and Demography, Faculty of Public Health, Khon Kaen University, Khon Kaen, Thailand; 2 Department of Epidemiology, Faculty of Public Health, Khon Kaen University, Khon Kaen, Thailand; 3 Clinical Trial Service Unit and Epidemiological Studies Unit, University of Oxford, Oxford, United Kingdom; 4 Cancer Unit, Faculty of Medicine, Khon Kaen University, Khon Kaen, Thailand

**Keywords:** alcohol consumption, mortality, health behaviour, rural population

## Abstract

**Background:**

The prevalence of alcohol consumption among Thais is high, around 30%. We quantified the relationship between alcohol drinking and mortality in a rural population in the most populous region of Thailand.

**Methods:**

The data were from the Khon Kaen Cohort Study. About 24 000 Thai adults were enrolled between 1990 and 2001, and follow-up for vital status continued until March 16, 2012. Mortality data were obtained from the Bureau of Policy and Strategy, Ministry of the Interior, Thailand. A Cox proportional hazards model was used to analyze the association between alcohol drinking and death, controlling for age, education level, and smoking, and floating absolute risk was used to estimate the 95% confidence intervals of hazard ratios.

**Results:**

In total, 18 457 participants (5829 men and 12 628 women) were recruited, of whom 3155 died (1375 men and 1780 women) during a median follow-up period of 13.6 years. Although alcohol drinking was common (64% of men and 25% of women), the amounts consumed were very low (average, 4.3 g/day in men and 0.8 g/day in women). As compared with never drinkers, mortality risk was lower among current drinkers and higher among ex-drinkers. Current drinking was not associated with mortality from cancer or diseases of the circulatory system, although ex-drinkers appeared to have a higher risk of death from the latter.

**Conclusions:**

The leading causes of mortality were not associated with current alcohol drinking at the low consumption levels observed in this population.

## INTRODUCTION

A J-shaped association between alcohol consumption and all-cause mortality has been reported in several cohort studies, ie, relative risks were less than unity among light drinkers as compared with abstainers.^1^ Most earlier studies were conducted in Western countries, and the beneficial effect of moderate alcohol consumption was attributed to decreased mortality from cardiovascular diseases.^2^^–^^5^ However, the risk of malignant tumors of the oral cavity, pharynx, larynx, esophagus, large bowel, liver, and breast are causally related to consumption of alcoholic beverages, with no apparent protective effect at low levels of consumption.^6^^,^^7^ The precise effect on overall mortality is therefore likely to be influenced by the pattern of cause-specific mortality, as well as by other factors such as the type of alcoholic beverages consumed. In addition, genetic factors may influence alcohol metabolism in different populations.

The leading causes of death in the Thai general population are malignant neoplasms, heart disease, and hypertension with cerebrovascular disease^8^ (death rates in 2009: 88.3, 29.0, and 24.7 per 100 000, respectively),^9^ all of which are related to alcohol consumption. Drinking alcohol is an established part of Thai culture when celebrating special occasions, and some Thais believe that drinking alcohol helps to increase appetite.^10^ Despite public and media campaigns to reduce alcohol consumption, the number of drinkers has not decreased; the prevalence of alcohol consumption in adults was 32.2% (54.5% in men; 10.8% in women) in 2009.^11^ We investigated the association between alcohol consumption and mortality in a cohort of individuals drawn from rural communities in the populous northeast region of Thailand.

## METHODS

### Study population

During the period from 1990 through 2001, the Khon Kaen cohort study^12^ recruited 24 528 healthy adults, most aged 35 to 64 years, from villages in northeast Thailand, a relatively underdeveloped region of the country. All participants underwent a simple physical examination, completed an interviewer-administered questionnaire (including sections on lifestyle, habits, and diet), and donated blood specimens, which were processed and stored in a biological bank at −20°C. The questions on alcohol asked about usual consumption, in terms of beverage type, unit of consumption, and frequency. Participants were excluded if they were younger than 30 years or 70 years or older at the time of enrollment (*n* = 944), if they had no national identification number (*n* = 3723), or if alcohol drinking status (*n* = 554) or alcohol dose were unknown (*n* = 850). Of the 24 528 participants, 18 457 remained in the study, and all were followed-up for cause-specific mortality.

### Measurement

The study interview was conducted by trained staff and included a detailed history of tobacco and alcohol use, as well as collection of routine demographic data. Information on past medical history was not obtained. Height, weight, and blood pressure were measured. Questions on alcohol use included type of alcoholic beverage, frequency, and amount usually consumed during their lifetime since starting to drink alcohol (for current drinkers) and age at cessation (for former drinkers). The categories used for alcohol drinking status were never drinker (lifetime never drinker or drank for less than 1 year before the enrollment date), ex-drinker (drank for at least 1 year and quit drinking for at least 1 year before the enrollment date), and current drinker (drank for at least 1 year and either never quit drinking or quit drinking less than 1 year before the enrollment date). Dose (cc per day) was calculated by dividing the amount drunk in a week by 7, in a month by 30, and in a period longer than 1 month by 35. The amount of alcohol consumed by current drinkers, in grams per day, was determined by the formula: [alcohol by volume (%) × (volume in cc/1000)] × 8.^13^^,^^14^ Alcohol volume was defined as 5% for beer, 7% for *sato* (rice wine), 35% for red whisky, and 40% for white whisky.^14^

### Mortality follow-up

Information on deaths among cohort members between enrollment and the closing date (March 16, 2012) was collected from the register of deaths compiled by the Bureau of Policy and Strategy, Office of Permanent Secretary, Ministry of Interior.^15^ The national identification number was used as the key to link the death database with the Khon Kaen study cohort. A member of the staff at Srinagarind Hospital, Khon Kaen University, coded the cause of death text into ICD-10 code groups, including malignant neoplasms (C00–C97), diseases of the circulatory system (I00–I99), and other causes (excluding C00–C97 and I00–I99).

### Statistical analysis

A Cox proportional hazards model was used to estimate hazard ratios (HRs) for the association of alcohol consumption with all-cause and cause-specific mortality by age (30–39, 40–49, 50–59, 60–69 years), education level (uneducated or lower primary school level, upper primary school level or higher), and smoking (never, ex-, current) as covariates. Participants still alive at the end of follow up, and those who died from another cause, were censored. The method of Plummer^16^ was used to estimate the variance of the log risk in each of the 3 groups, and from these variances 95% CIs were derived for each group, including the reference group, to describe the effects of the play of chance, within that 1 group, on the HR. The actual regression estimates remain unchanged. Analysis using Cox proportional hazards models adjusted for age, education, and smoking was done with STATA version 10.

### Ethical considerations

This study was approved by the Khon Kaen University Ethics Committee for Human Research (Reference No. HE541374).

## RESULTS

**Figure 1.**
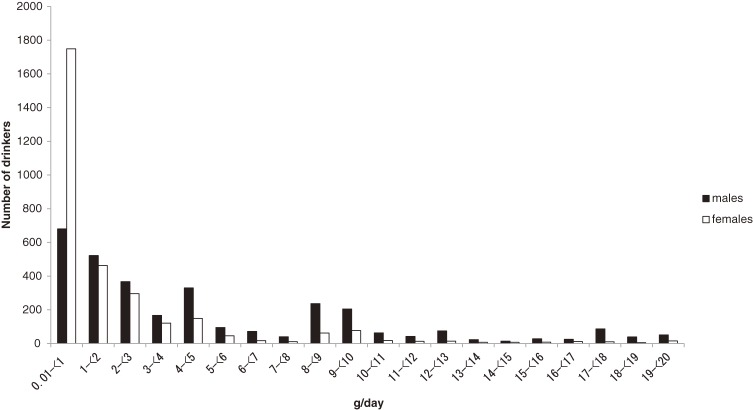
Number of current drinkers consuming less than 20 g/day, by amount of alcohol consumed daily (3162 men, 3101 women).

A total of 18 457 participants (5829 men, 12 628 women) aged 30 to 69 years were included in the study. The prevalence of alcohol drinking was higher among men than among women (64.2% and 25.3%, respectively; eTable). The proportion of current drinkers, and the amount consumed by current drinkers, decreased with increasing age in both sexes. Most drinkers consumed only modest amounts of alcohol: median alcohol consumption was 4.3 g/day in men and 0.8 g/day in women (eTable). Only 582 (15.5%) of male current drinkers, and just 94 (2.9%) of female current drinkers, consumed 20 g or more alcohol per day (Figure 1). Among male drinkers, the most popular beverage was white whisky (40.6%; median alcohol consumption, 2.7 g/day), followed by beer (29.5%; 1.0 g/day), red whisky (18.2%; 1.9 g/day), and *sato* (rice wine; 11.7%; 0.4 g/day).

After controlling for age, there was no association with all-cause mortality among male current drinkers, but there was an inverse association among female drinkers (eFigure 1). There was a difference in age between ex-drinkers and current drinkers (mean 53.7 vs 50.5 years in men; 51.7 vs 48.6 years in women; Table). The proportion of individuals with an upper primary school level education or higher (≥5 years of education) was larger among current drinkers than among ex-drinkers (14.0% and 11.5%, respectively, in men; 6.7% and 0.6% in women). Among men, 44.2% of never drinkers and 65.5% of current drinkers were current smokers. Age, social status, and smoking were important determinants of mortality in our cohort.^17^ Accordingly, models of the association between alcohol drinking and mortality were adjusted for these 3 variables.

**Table.** Baseline characteristics of participants by alcohol drinking category (5829 men, 12 628 women)

Characteristics	Never drinkers	Ex-drinkers	Current drinkers	Never drinkers	Ex-drinkers	Current drinkers
	
	Men			Women		
	
Age, years	53.1 (8.8)	53.7 (8.3)	50.5 (8.1)	50.9 (8.9)	51.7 (7.9)	48.6 (7.8)
BMI, kg/m^2^ (missing, 30.1%)	22.8 (3.3)	22.7 (3.1)	22.6 (3.0)	24.3 (4.0)	24.2 (3.8)	24.4 (3.9)
SBP, mm Hg (missing, 30.2%)	118.7 (15.9)	119.3 (15.4)	117.8 (14.0)	119.3 (15.9)	118 (15.0)	117.8 (14.3)
DBP, mm Hg (missing, 30.2%)	77.9 (10.7)	78.0 (10.0)	77.5 (10.1)	77.7 (10.5)	76.2 (10.4)	77.0 (10.0)
Residence: municipal area, *n* (%)	306 (20.0)	114 (20.5)	795 (21.2)	2031 (21.9)	33 (18.6)	713 (22.3)
Education (missing, 2.4%): upper primary school level or higher, *n* (%)	208 (14.0)	62 (11.5)	513 (14.0)	437 (4.8)	1 (0.6)	207 (6.7)
Smoking (missing, 4.4%)						
Never smokers, *n* (%)	602 (41.0)	60 (11.1)	628 (17.6)	8973 (99.5)	169 (98.3)	3018 (97.8)
Ex-smokers, *n* (%)	216 (14.7)	238 (44.2)	602 (16.9)	5 (0.1)	2 (1.2)	9 (0.3)
Current smokers, *n* (%)	649 (44.2)	241 (44.7)	2336 (65.5)	38 (0.4)	1 (0.6)	59 (1.9)

Abbreviations: BMI, body mass index; DBP, diastolic blood pressure; SBP, systolic blood pressure.

Values are mean (SD).

**Figure 2.**
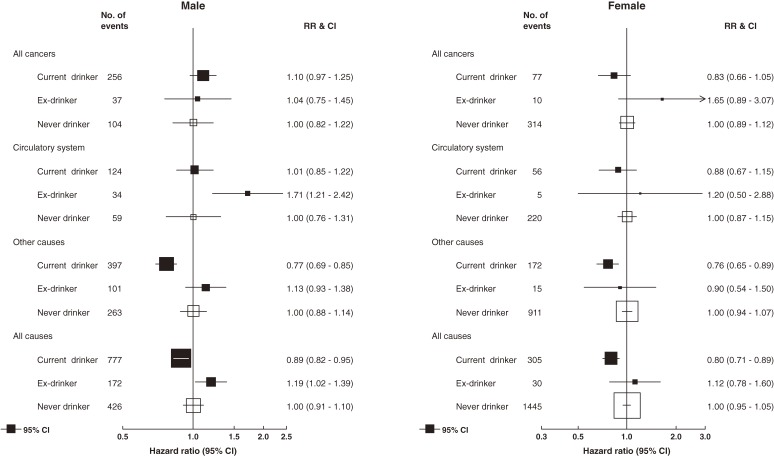
Hazard ratios for disease-specific and all-cause mortality among current and ex-drinkers as compared with never drinkers, adjusted for age, education level, and smoking (men = 5829, women = 12 628). Person-years of follow-up by alcohol drinking status: men—current = 51 816, ex = 7509, and never = 21 391; women—current = 44 447, ex = 2495, and never = 132 624.

After a median follow-up period of 13.6 years (range, 0.02–22.2 years), there were 3155 deaths among cohort members (1375 men, 1780 women). Ex-drinkers had the highest crude mortality rate in both sexes (22.9 and 12.0 per 1000 per year in men and women, respectively). Mortality among never drinkers (19.9 and 10.9 per 1000 per year in men and women, respectively) was higher than that among current drinkers (15.0 and 6.9 per 1000 per year in men and women, respectively). Figure 2 shows the HRs for all-cause and cause-specific mortality among male and female current drinkers and ex-drinkers, as compared with never drinkers. All-cause mortality was lower in current drinkers than in never drinkers in both sexes, although, in the analysis of specific causes, this pattern was observed only for the category “other diseases”; the differences for cancer and diseases of the circulatory system were not significant.

As compared with male never drinkers, male ex-drinkers had significantly higher risks of death from any cause (HR 1.19; 95% CI, 1.02–1.39) and death from diseases of the circulatory system (HR 1.71; 95% CI, 1.21–2.42) but not death from cancer or other causes. There were too few female ex-drinkers (30 deaths) for reliable analyses. Excluding deaths within 1 year and 3 years of enrollment into the cohort did not change these risk patterns among ex-drinkers (eFigure 2).

## DISCUSSION

Numerous studies have investigated the effects of alcohol consumption on overall (all-cause) and disease-specific mortality, including several studies of Asian populations. Almost all found that the relationship with all-cause mortality is J-shaped: mortality was lower among light/moderate drinkers than among never drinkers, although associations with specific diseases were more variable.

This is the first community-based cohort study in Thailand. It had a long follow-up period (median, 13.6 years) and enrolled a purely rural population. The prevalence of alcohol consumption among cohort members was relatively high (almost two-thirds of men and one-quarter of women) as compared with the national smoking and alcohol drinking survey of 2007 (54% in men and 11% in women aged 50–54 years), although alcohol drinking was about 25% more frequent in northeast Thailand as compared with the country overall.^18^ However, the amount of alcohol consumed by drinkers was very small (median, 4.3 g/day in men and 0.8 g/day in women) and lower than that in the national survey (8.8 g/day in men, and 1.9 g/day in women, aged 45–65 years).^19^ Very few members of the population consumed more than a very modest amount of alcohol (only 15.5% of male drinkers and <2.9% of female drinkers consumed at least 140 g/week). In contrast, 81% of drinkers in a Chinese cohort^20^ and 78% of drinkers in a Japanese cohort^21^ consumed more than 140 g/week.

The low all-cause mortality among light drinkers in our cohort is entirely consistent with the results of other studies,^22^ including those conducted among Asian populations in China,^20^ Japan,^21^^,^^23^^,^^24^ and Korea.^25^ We did not observe any significant association between modest alcohol drinking and mortality from either cancers or diseases of the circulatory system. In general, previous studies found that light drinkers had a lower risk of mortality from ischemic heart disease but not from stroke,^26^^,^^27^ and deaths from cerebrovascular disease (*n* = 76) considerably exceeded those from ischemic heart disease (*n* = 57) in our cohort. Moreover, other studies in Asia found no protective effect against ischemic heart disease.^20^^,^^28^ With respect to cancer, there is no reason to expect a decrease in risk at any level of alcohol consumption, and, given that alcohol increases the risk of several cancers (eg, cancers of the oral cavity, pharynx, esophagus, and larynx), mortality might actually be increased, even among light drinkers. However, in the Khon Kaen population, the most common causes of cancer mortality in men are cancer of the liver and intrahepatic bile ducts (predominantly cholangiocarcinoma, which is unrelated to alcohol intake), lung, and colon, so the lack of a clear relation to alcohol is unsurprising.

“Other causes” of death was a very heterogeneous group in this study; however, some of the included causes—eg, respiratory diseases (5.1%)—were associated with lower mortality rates among light drinkers.^5^^,^^20^^,^^29^

Among males, ex-drinkers were more likely to die than never drinkers, and this was particularly clear for deaths due to diseases of the circulatory system. This has been observed in other prospective studies.^3^^,^^30^^–^^32^ An obvious explanation is reverse causality (individuals with fatal illnesses may have already stopped drinking alcohol at the time they were enrolled into the cohort). However, this possibility was minimized by restricting cohort recruitment to apparently healthy individuals.^12^ In addition, exclusion of deaths occurring up to 3 years after enrollment did not diminish the excess risk of death. Nevertheless, this possibility cannot be entirely excluded. In a study of British doctors, Doll et al (2005)^5^ found that excess risk was confined to recent ex-drinkers and that the risk of death among those who had quit alcohol 13 or more years earlier was similar to that of never drinkers.

The strengths of our study are its prospective design, the relatively long follow-up period (median duration of follow-up, 13.6 years), the distinction between ex-drinkers and current drinkers, and the availability of information on amount and frequency of alcohol consumption and important confounding variables—especially smoking—which allowed for appropriate adjustment in the analysis. In addition, outcome information—death data from a national mortality database complied by the Ministry of the Interior—was unbiased by exposure.

The study limitations include the fact that alcohol consumption data were collected by questionnaire, with no means of validating participant responses (in many countries, alcohol consumption is known to be systematically underestimated by questionnaire). The analysis was restricted to data on consumption at enrollment—it is possible that some current drinkers became abstainers during the follow-up period (it is less likely that lifelong abstainers would begin drinking). Cause of death was certified by a physician in around 75% of cases, but a large proportion of deaths (24.4%) were recorded in the R section of ICD-10 (senility and ill-defined causes), and the validity of recorded cause is uncertain.^33^^,^^34^ The relationship between alcohol drinking and mortality due to transport accident could not be analyzed because of the very small number of deaths (*n* = 13), although this cause of death ranks second in Thailand and was found to be associated with alcohol consumption in previous studies.^35^^–^^37^ Finally, as indicated above, we were unable to fully analyze the association between amount of alcohol consumed and mortality because very few of the study participants consumed more than a small amount of alcohol. However, drinking small amounts of alcohol may have a protective effect on health. Data on history of illness should be collected in future research.

In conclusion, among men there was a weak but statistically significant association between current alcohol drinking and all-cause mortality. The associations between current alcohol consumption and cause-specific mortality due to cancers or diseases of the circulatory system were not statistically significant. The pattern was similar among women: current drinking was associated with all-cause mortality but not with cancers or diseases of the circulatory system. The leading causes of mortality were not associated with current alcohol drinking at the low consumption levels observed in this study.

## ONLINE ONLY MATERIALS

eTable. Prevalence of alcohol drinking by age group (5829 men, 12 628 women).

eFigure 1. Age-adjusted hazard ratios for disease-specific and all-cause mortality among current and ex drinkers as compared with never drinkers (men = 5829, women = 12 628).

eFigure 2. Hazard ratios for disease-specific and all-cause mortality among ex-drinkers as compared with never drinkers, adjusted for age, education level, and smoking. Model 1: All subjects (men = 5829; women = 12 628). Model 2: Deaths within first year of follow-up excluded (men = 5780; women = 12 591). Model 3: Deaths within first 3 years of follow-up excluded (men = 5670; women = 12 473).
