# Supplementary material for: Alcohol Consumption and Mortality: The Khon Kaen Cohort Study, Thailand
Source: J Epidemiol. 2014 Mar 5;24(2):154–60. doi: 10.2188/jea.JE20130092 (PMC3956694; doi:10.2188/jea.JE20130092)
Supplement: eTable. [file je-24-154-s001.pdf]

eTable. Prevalence of alcohol drinking by age group (5,829 men, 12,628 women)

Age (years)	Men						
	Never drinkers		Ex-drinkers		Current drinkers		
	n	%	*n*	%	*n*	%	g/ day
30 – 39	105	21.8	32	6.7	344	72	6.5
40 – 49	437	22.2	148	7.5	1,382	70	4.6
50 – 59	552	25.0	218	9.9	1,435	65	4.0
60 – 69	435	37.0	158	13.4	583	50	3.1
All	1,529	26.2	556	9.5	3,744	64	4.3

Age (years)	Women						
	Never drinkers		Ex-drinkers		Current drinkers		
	n	%	*n*	%	*n*	%	g/ day
30 – 39	1,122	71.7	12	0.8	431	27.5	1.2
40 – 49	2,981	67.8	63	1.4	1,352	30.8	0.8
50 – 59	3,325	74.1	71	1.6	1,089	24.3	0.6
60 – 69	1,828	83.8	31	1.4	323	14.8	0.5
All	9,256	73.3	177	1.4	3,195	25.3	0.8
