# Supplementary material for: Alcohol Consumption and Mortality: The Khon Kaen Cohort Study, Thailand
Source: J Epidemiol. 2014 Mar 5;24(2):154–60. doi: 10.2188/jea.JE20130092 (PMC3956694; doi:10.2188/jea.JE20130092)
Supplement: eFigure 1. [file je-24-154-s002.pdf]

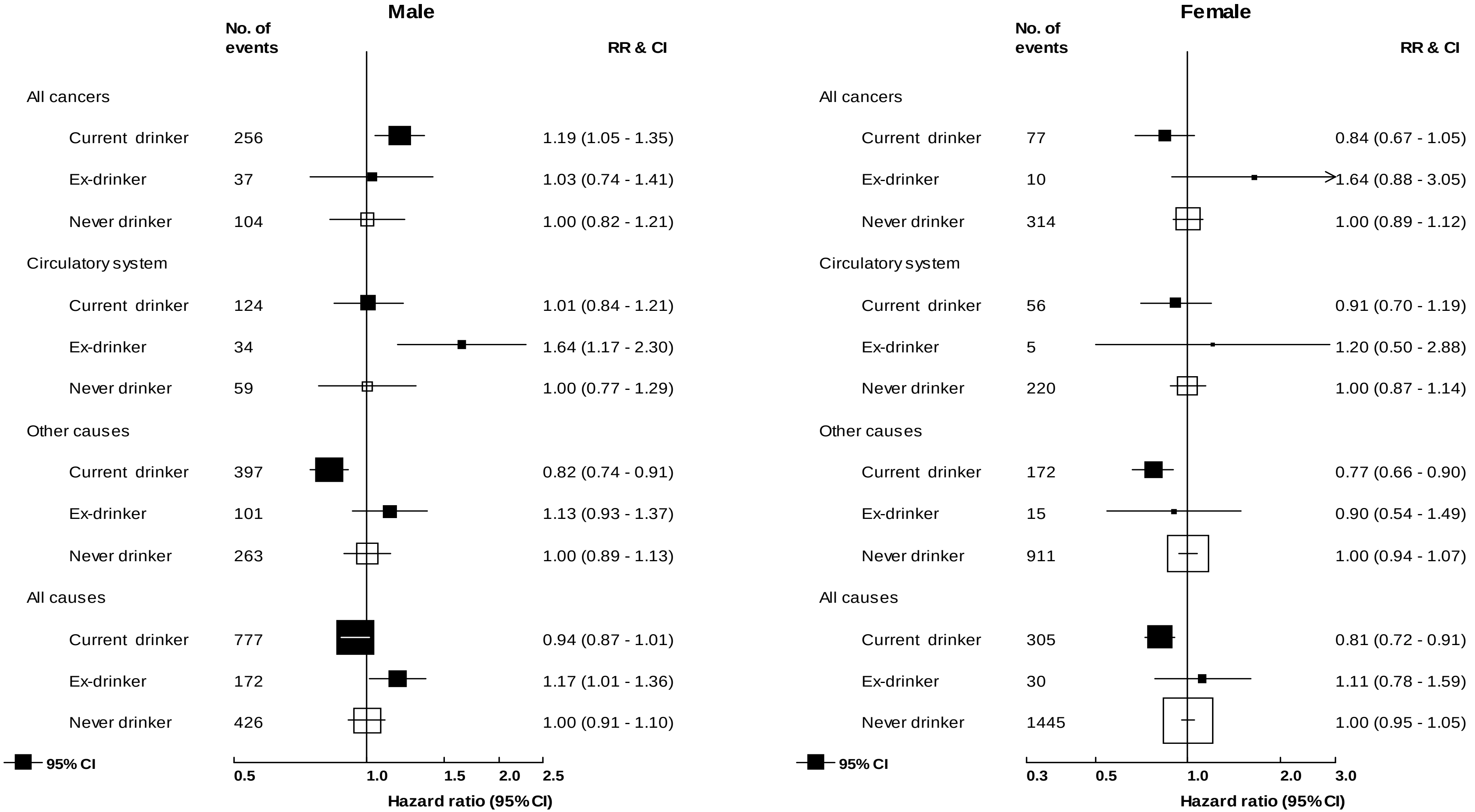

eFigure 1. Age-adjusted hazard ratios for disease-specific and all-cause mortality among current and ex drinkers as compared with never drinkers (men=5,829, women=12,628)
