# Supplementary material for: Alcohol Consumption and Mortality: The Khon Kaen Cohort Study, Thailand
Source: J Epidemiol. 2014 Mar 5;24(2):154–60. doi: 10.2188/jea.JE20130092 (PMC3956694; doi:10.2188/jea.JE20130092)
Supplement: eFigure 2. [file je-24-154-s003.pdf]

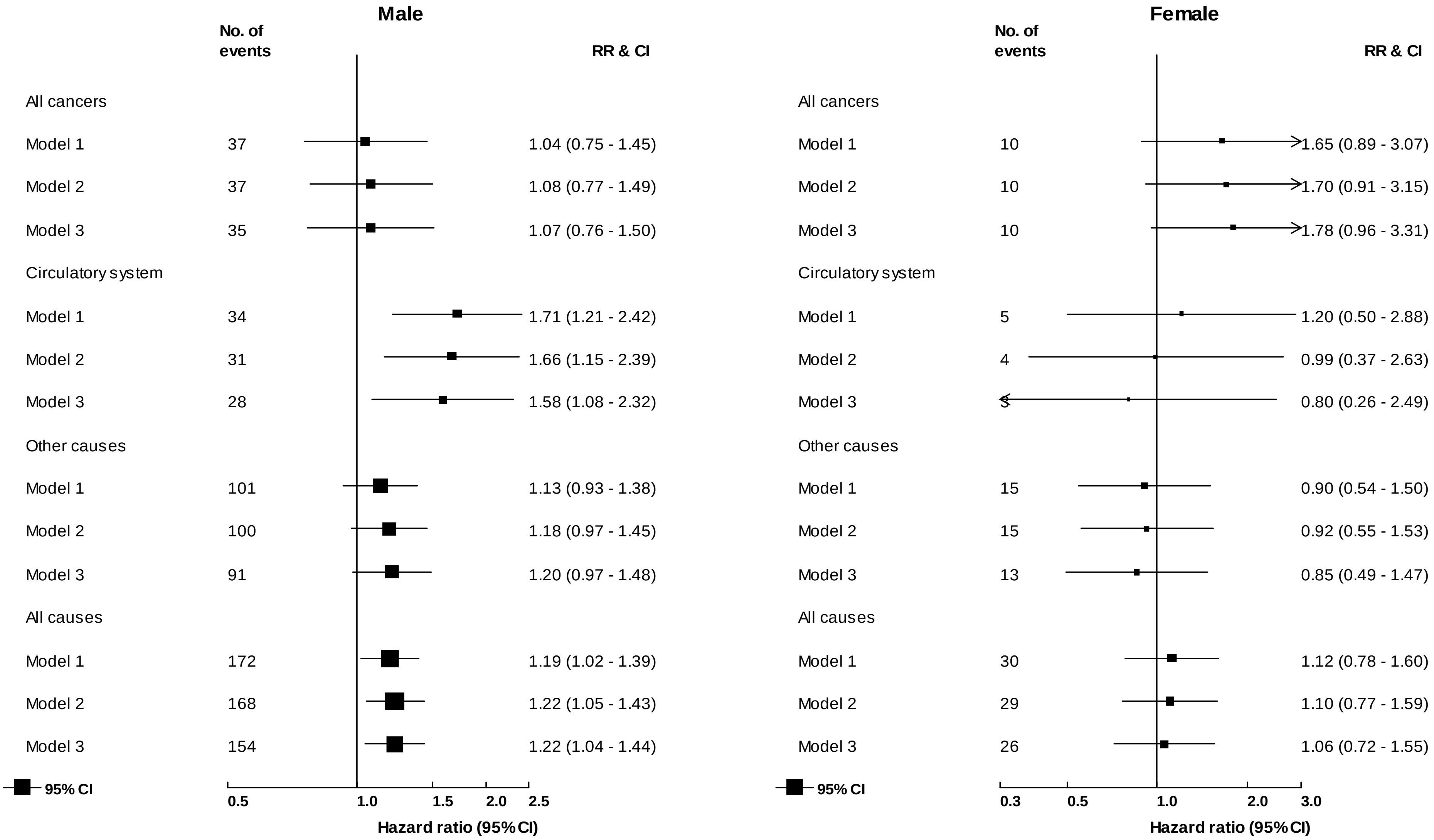

eFigure 2. Hazard ratios for disease-specific and all-cause mortality among ex-drinkers as compared with never drinkers, adjusted for age, education level, and smoking

Model 1: All subjects (men=5,829; women=12,628)

Model 2: Deaths within first year of follow-up excluded (men=5,780; women=12,591)

Model 3: Deaths within first 3 years of follow-up excluded (men=5,670; women=12,473)
